# Oxidized organic molecules in the tropical free troposphere over Amazonia

**DOI:** 10.1093/nsr/nwad138

**Published:** 2023-05-15

**Authors:** Qiaozhi Zha, Diego Aliaga, Radovan Krejci, Victoria A Sinclair, Cheng Wu, Giancarlo Ciarelli, Wiebke Scholz, Liine Heikkinen, Eva Partoll, Yvette Gramlich, Wei Huang, Markus Leiminger, Joonas Enroth, Otso Peräkylä, Runlong Cai, Xuemeng Chen, Alkuin Maximilian Koenig, Fernando Velarde, Isabel Moreno, Tuukka Petäjä, Paulo Artaxo, Paolo Laj, Armin Hansel, Samara Carbone, Markku Kulmala, Marcos Andrade, Douglas Worsnop, Claudia Mohr, Federico Bianchi

**Affiliations:** Institute for Atmospheric and Earth System Research / Physics, University of Helsinki, Helsinki00014, Finland; Joint International Research Laboratory of Atmospheric and Earth System Sciences, Nanjing University, Nanjing210023, China; Institute for Atmospheric and Earth System Research / Physics, University of Helsinki, Helsinki00014, Finland; Department of Environmental Science & Bolin Centre for Climate Research, Stockholm University, Stockholm, SE-106 91, Sweden; Institute for Atmospheric and Earth System Research / Physics, University of Helsinki, Helsinki00014, Finland; Department of Chemistry and Molecular Biology, University of Gothenburg, Gothenburg 40530, Sweden; Institute for Atmospheric and Earth System Research / Physics, University of Helsinki, Helsinki00014, Finland; Institute for Ion and Applied Physics, University of Innsbruck, Innsbruck 6020, Austria; Institute for Atmospheric and Earth System Research / Physics, University of Helsinki, Helsinki00014, Finland; Department of Environmental Science & Bolin Centre for Climate Research, Stockholm University, Stockholm, SE-106 91, Sweden; Institute for Ion and Applied Physics, University of Innsbruck, Innsbruck 6020, Austria; Department of Environmental Science & Bolin Centre for Climate Research, Stockholm University, Stockholm, SE-106 91, Sweden; Institute for Atmospheric and Earth System Research / Physics, University of Helsinki, Helsinki00014, Finland; Institute for Ion and Applied Physics, University of Innsbruck, Innsbruck 6020, Austria; Ionicon Analytik GmbH, Innsbruck 6020, Austria; Institute for Atmospheric and Earth System Research / Physics, University of Helsinki, Helsinki00014, Finland; Institute for Atmospheric and Earth System Research / Physics, University of Helsinki, Helsinki00014, Finland; Institute for Atmospheric and Earth System Research / Physics, University of Helsinki, Helsinki00014, Finland; Institute for Atmospheric and Earth System Research / Physics, University of Helsinki, Helsinki00014, Finland; Laboratory for Atmospheric Physics, Institute for Physics Research, Universidad Mayor de San Andrés, La Paz, Bolivia; Laboratory for Atmospheric Physics, Institute for Physics Research, Universidad Mayor de San Andrés, La Paz, Bolivia; Laboratory for Atmospheric Physics, Institute for Physics Research, Universidad Mayor de San Andrés, La Paz, Bolivia; Institute for Atmospheric and Earth System Research / Physics, University of Helsinki, Helsinki00014, Finland; Institute of Physics, University of Sao Paulo, Sao Paulo 05508-900, Brazil; Institute for Atmospheric and Earth System Research / Physics, University of Helsinki, Helsinki00014, Finland; Institute for Geosciences and Environmental Research (IGE), University of Grenoble Alpes, Grenoble38000, France; Institute for Ion and Applied Physics, University of Innsbruck, Innsbruck 6020, Austria; Agrarian Sciences Institute, Federal University of Uberlândia, Uberlândia 38408-100, Brazil; Institute for Atmospheric and Earth System Research / Physics, University of Helsinki, Helsinki00014, Finland; Joint International Research Laboratory of Atmospheric and Earth System Sciences, Nanjing University, Nanjing210023, China; Beijing Advanced Innovation Center for Soft Matter Science and Engineering, Beijing University of Chemical Technology, Beijing100029, China; Laboratory for Atmospheric Physics, Institute for Physics Research, Universidad Mayor de San Andrés, La Paz, Bolivia; Department of Atmospheric and Oceanic Sciences, University of Maryland, College Park, MD 20742, USA; Institute for Atmospheric and Earth System Research / Physics, University of Helsinki, Helsinki00014, Finland; Aerodyne Research, Inc., Billerica, MA01821, USA; Department of Environmental System Science, ETH Zürich, Zürich 8092, Switzerland; Switzerland and Laboratory of Atmospheric Chemistry, Paul Scherrer Institute, Villigen 5232, Switzerland; Institute for Atmospheric and Earth System Research / Physics, University of Helsinki, Helsinki00014, Finland

**Keywords:** oxidized organic molecules, tropical aerosol formation, Amazon, free troposphere

## Abstract

New particle formation (NPF) in the tropical free troposphere (FT) is a globally important source of cloud condensation nuclei, affecting cloud properties and climate. Oxidized organic molecules (OOMs) produced from biogenic volatile organic compounds are believed to contribute to aerosol formation in the tropical FT, but without direct chemical observations. We performed *in situ* molecular-level OOMs measurements at the Bolivian station Chacaltaya at 5240 m above sea level, on the western edge of Amazonia. For the first time, we demonstrate the presence of OOMs, mainly with 4–5 carbon atoms, in both gas-phase and particle-phase (in terms of mass contribution) measurements in tropical FT air from Amazonia. These observations, combined with air mass history analyses, indicate that the observed OOMs are linked to isoprene emitted from the rainforests hundreds of kilometers away. Based on particle-phase measurements, we find that these compounds can contribute to NPF, at least the growth of newly formed nanoparticles, in the tropical FT on a continental scale. Thus, our study is a fundamental and significant step in understanding the aerosol formation process in the tropical FT.

## Teaser

In-situ molecular-level measurements demonstrate the presence of oxidized organic molecules, mainly with 4-5 carbon atoms, in both gas-phase and particle-phase in tropical free troposphere air from Amazonia. These molecules are linked to isoprene emitted from the rainforests hundreds of kilometers away, and can contribute to new particle formation.

## INTRODUCTION

The tropical free troposphere (FT) can host large numbers of aerosol particles, serving as cloud condensation nuclei (CCN), and thus affects the climate system on a global scale [1,2]. Atmospheric new particle formation (NPF) comprises two crucial steps: nucleation and subsequent growth of newly formed nanoparticles. NPF has been consistently observed in the tropical FT in regions with high concentrations (in terms of particle number) of ultrafine particles (UFPs, here defined as particles with diameters of between 10 and 50 nm) using an aircraft and is thus likely to be a significant source of FT aerosols [1–4]. Oxidized organic molecules (OOMs), produced from biogenic volatile organic compounds (BVOCs) carried up to the FT by mesoscale convective systems, are hypothesized to be key components in forming aerosols due to their reduced volatility at low temperatures [1,2,4–6].

Recent modeling studies have reported that biogenic-related NPF likely dominates FT aerosol formation in tropical BVOC emission hotspots such as Amazonia [6–8]. Elucidating the chemical composition of OOMs is required to constrain model simulations and improve understanding of the mechanism and influence of biogenic-related NPF in the tropical FT [7]. Direct observations to date are limited to airborne studies lacking necessary chemistry instrumentation [1–4]. The Southern hemisphere high ALTitude Experiment on particle Nucleation And growth (SALTENA) campaign (see [9] and ‘Methods’) performed direct molecular-level observations of OOMs using a set of state-of-the-art mass spectrometers at the Bolivian Global Atmosphere Watch (GAW) station Chacaltaya (CHC; 5240 m above sea level (a.s.l.)), on the western edge of the Amazon Basin (Supplementary Fig. S1). These measurements complemented long-term observations at CHC of e.g. particle number size distribution and equivalent black carbon (eBC). For this campaign, we reconstructed the 96-h air mass history using the Lagrangian particle dispersion model FLEXPART-WRF to determine the origin and footprint of air masses arriving at CHC (see [10] and ‘Methods’).

## RESULTS

The present study focuses on measurements in January 2018 during the austral summer (wet season), when FT air from Amazonia exerted a large influence on CHC compared with other periods of the campaign (‘Methods’). The data were divided into FT, mixed FT and local air (non-FT), and daytime events. The FT events occurred during night-time (19 : 00–06 : 00; local time, UTC–4) when the influence of FT air was dominant at CHC. These periods were characterized by low water vapor mixing ratio (WVMR) and eBC concentrations, as tropical FT air is typically drier and less polluted (here indicated by the lower eBC concentration) than the boundary layer (BL) air during the wet season (see ‘Methods’ for the detailed description of FT event identification) [11–13]. The remaining night-time periods were defined as non-FT periods when the influence of local BL air was more significant compared with FT periods. Due to the thermally driven local mixing layer cycle and air circulation, the impact of BL air was evident at CHC from 07 : 00 to 18 : 00. As a result, during daytime periods, CHC was impacted by urban air pollution from the nearby La Paz—El Alto metropolitan area [11,12].

**Figure 1.**
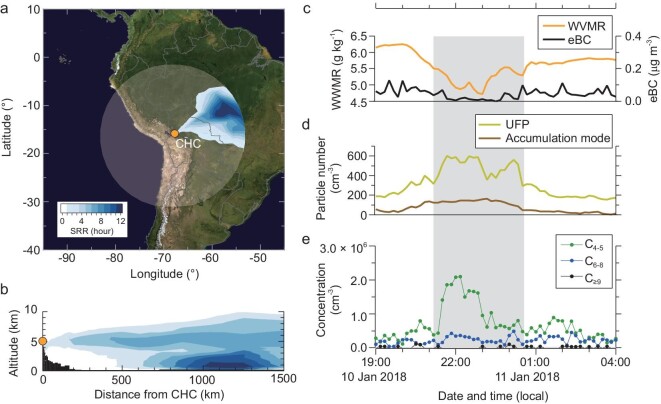
An Amazon FT event observed at CHC in the night of 10 January 2018. (a) Horizontal profile of the vertically integrated source receptor relationship (SRR, units of hours) derived from the FLEXPART-WRF model and averaged from 21 : 00 to 00 : 00 (local date and time, UTC–4). The semi-transparent circle indicates the horizontal output domain of the model. The model output is in 1-h time resolution. The color bar denotes the SRR values of the passive air tracers. (b) Vertical profile of the SRR integrated over the radial direction, averaged from 21 : 00 to 00 : 00. Black shading indicates the topography near the station. (c) Concentrations of WVMR and eBC. The gray shaded area denotes the exact period of the FT event, identified with WVMR ≤ 5.5 g kg^−1^ and eBC ≤ 0.08 μg m^−3^ (‘Methods’). (d) Concentrations of UFPs (diameter of 10–50 nm) and accumulation mode particles (diameter of 100–500 nm). (e) Concentrations of oxidized organic molecules (OOMs) measured by using a nitrate-based CI-APi-TOF; grouped based on their number of carbon atoms (C_4__–__5_, C_6__–__8_ and C_≥9_). The C_≥9_ OOM concentration is below the detection limit of the CI-APi-TOF (‘Methods’) for most of the time during this FT event.

During the study period, FT events at CHC were frequently observed (on 14 out of 17 nights; Supplementary Fig. S2) under the persistent influence of air masses from Amazonia. An FT event is exemplarily shown in Fig. 1 for the night of 10 January 2018 (from 21 : 10 to 00 : 30), when very low WVMR (4.7 g kg^−1^) and eBC concentrations (close to the detection limit) were measured. Detailed, high-resolution analysis of the modeled source-receptor relationship (SRR; see [10] and ‘Methods’) for this event, which links the sampled air masses at CHC via their transport and residence time therein, shows the Amazon Basin as the origin (Fig. 1a and Supplementary Fig. S3, with hourly air mass history from 19 : 00 to 03 : 00). The SRR vertical profile (Fig. 1b) further shows that a large fraction of these air masses had spent considerable time within the Amazon BL in the region of 800–1400 km away from CHC before ascending to the FT. Similar to all other FT events in January 2018, MODIS (Moderate Resolution Image Spectroradiometer) satellite images show that several mesoscale convective systems were present concurrently over this region (Supplementary Figs S4 and S5), indicating that air masses from the Amazon BL were transported to the tropical FT through convective lifting. Once in the FT, the air masses were transported via horizontal advection to CHC in ≤36 h. In this way, the Amazon Basin can continuously and widely affect the tropical FT during the wet season.

The composition of FT air from Amazonia showed notable differences from that during non-FT periods on the night of 10 January 2018. WVMR and eBC concentrations started to decrease at 20 : 00 and reached a minimum after ∼1 h, indicating that by 21 : 10, the CHC was dominantly influenced by FT air (Fig. 1c). The FT condition persisted for 3 h until shortly after midnight on 11 January 2018. Figure 1d shows increased UFP concentrations during that period. The hourly averaged particle size spectra (Supplementary Fig. S6) show that the increase in UFPs was primarily due to enhancement of particles with diameters of between 20 and 30 nm. This is consistent with particle size spectra measured in the Amazon FT using an aircraft [3,4]. We divide observed gas-phase OOMs (Fig. 1e) measured by a nitrate-based chemical ionization atmospheric pressure interface time-of-flight mass spectrometer (CI-APi-TOF) into three groups based on their number of carbon atoms (C_4__–5_, C_6__–8_ and C_≥9_). Whereas C_6__–8_ and C_≥9_ OOM concentrations remained at low levels throughout the night, C_4__–5_ OOM concentrations increased during the FT event. A concurrent increase in the total signal of organic ions was also measured by using an APi-TOF (Supplementary Fig. S7), as compositions of OOMs and organic ions (negative ion adducts of OOMs) were usually identical [14,15]. More FT events are shown in Supplementary Figs S3, S8 and S9.

**Figure 2.**
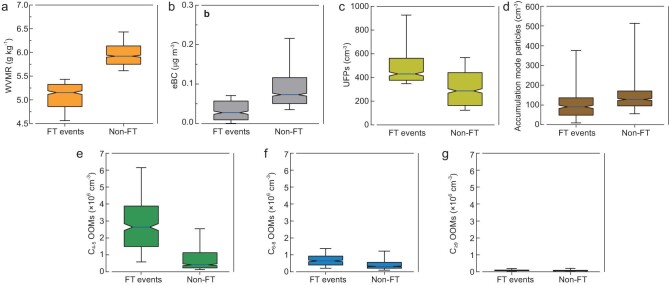
Variation in the parameters measured during periods with FT events and without FT events (non-FT). Only night-time data during the study period (January 2018) are included. (a) WVMR concentrations. (b) eBC mass concentrations. (c) Number concentrations of UFPs. (d) Number concentrations of accumulation mode particles. (e) Concentrations of C_4__–5_ OOMs. (f) Concentrations of C_6__–8_ OOMs. (g) Concentrations of C_≥9_ OOMs. Concentrations of C_≥9_ OOMs were close to the detection limit during the study period. OOMs with fewer than four carbon atoms (not shown here) were mostly composed of small organic acids, including malonic acid (C_3_H_4_O_4_) and oxalic acid (C_2_H_2_O_4_), which could come from both biogenic and anthropogenic sources. Boxes and whiskers are plotted for the 10th, 25th, 50th, 75th and 90th percentiles. Notches denote the 95% confidence interval of the median value. The number of data points (10-min resolution) for FT events and non-FT periods are 370 and 842, respectively. Panels (f) and (g) with *y*-axis on smaller scales are shown in Supplementary Fig. S10.

Overall, the characteristics determined based on all FT events are distinct from those of non-FT periods. WVMR and eBC concentrations (Fig. 2a and b) were significantly lower during FT conditions compared with non-FT conditions. High number concentrations of UFPs (Fig. 2c and d) are another typical feature of Amazon FT air, which is attributed to the significantly different properties between particles in the Amazon FT and BL. Previous aircraft-based aerosol studies showed that FT particles are predominately Aitken-mode particles and are barely affected by particles from the Amazon BL [1,3,4]. Concentrations of C_6__–8_ and C_≥9_ OOMs were much lower than C_4__–5_ OOM concentrations (Fig. 2e–g) and showed no clear differences between FT events and non-FT periods for these two groups. In contrast, C_4__–5_ OOM concentrations in FT events were significantly higher than that during non-FT periods, indicating that tropical FT air is enriched in C_4__–5_ OOMs during the wet season.

**Figure 3.**
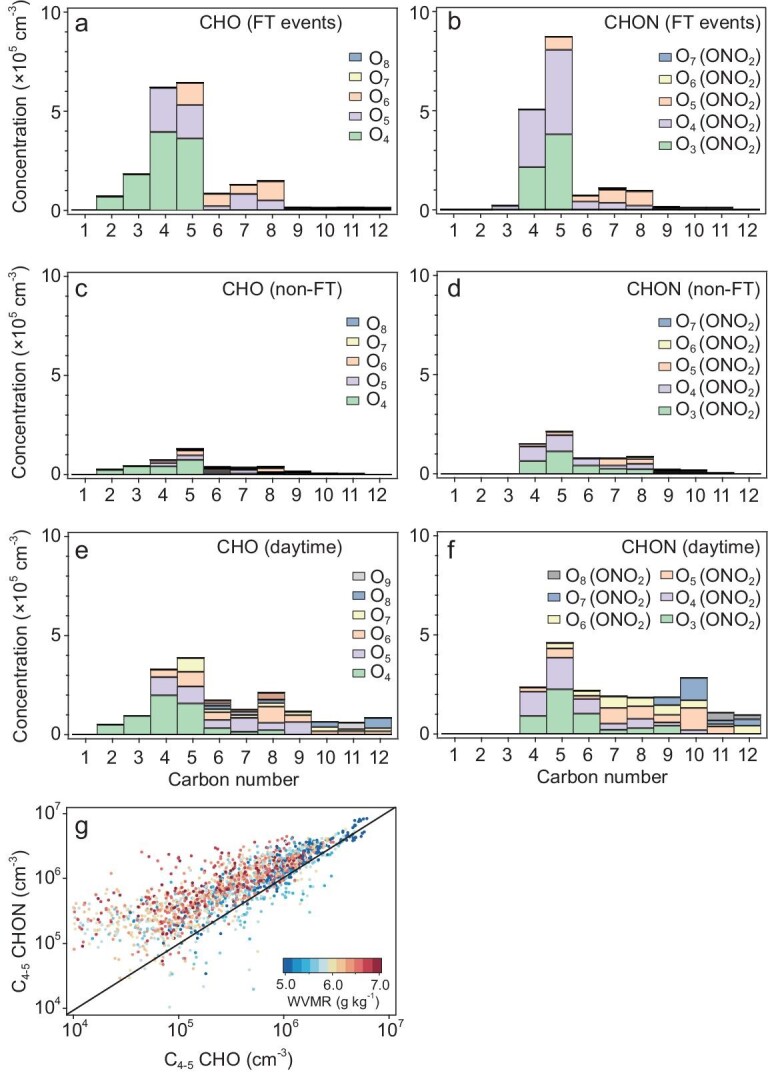
Chemical composition of OOMs observed in different conditions at CHC in January 2018. (a) and (b) Concentrations of OOMs containing (a) carbon, hydrogen and oxygen atoms (CHO) and (b) carbon, hydrogen, oxygen and nitrogen atoms (CHON) averaged over all FT events. OOMs are grouped with different oxygen atom numbers as a function of carbon atom numbers. Note that the contribution of a nitrate (-ONO_2_) functional group to OOM volatility is comparable to an alcohol (-OH) group due to the same effective O : C ratio [29]. (c) and (d) Concentrations of (c) CHO and (d) CHON OOMs averaged over ‘non-FT’ periods during night-time (19 : 00–06 : 00). (e) and (f) Concentrations of (e) CHO and (f) CHON OOMs averaged over daytime (07 : 00–18 : 00) when CHC was affected by BL air from the nearby La Paz—El Alto metropolitan area. (g) Correlation between C_4-5_ CHO and C_4-5_ CHON in January 2018 (including both daytime and night-time data), colored by the WVMR. Here the FT events are indicated by lower WVMR and higher OOM concentrations.

We further compare the molecular composition of OOMs during FT, non-FT and daytime events (Fig. 3). The variability in OOM composition among these periods strongly indicates different sources, pathways and origins of OOMs observed at CHC. During FT events, C_4__–5_ OOMs such as C_4_H_6,8_O_4,5_ and C_5_H_6,8,10_O_4,5_ were the dominant CHO species (Fig. 3a). These compounds have been observed previously in chamber studies on the oxidation of isoprene (C_5_H_8_) by hydroxyl radicals (OH) [16,17]. The most abundant CHONs (Fig. 3b) were C_4_H_7,9_O_3,4_(ONO_2_) and C_5_H_7,9_O_3,4_(ONO_2_), which have been characterized as OH-oxidation products of isoprene in the presence of NO_x_ (NO_x_ = NO + NO_2_) [17–19]. C_6__–8_ OOMs observed in FT events consisted of several compounds, mainly C_6_H_10_O_6_, C_7_H_9_,_11_O_5_(ONO_2_) and C_8_H_12,14_O_6_, which could be from various sources, such as the residual of daytime BL air, evaporation from existing particles and oxidation products of other BVOCs (e.g. monoterpenes). Furthermore, the composition of OOMs in the FT air from the Amazon Basin differs significantly from that in other regions. OOMs observed in the FT over other regions often contain a higher number of carbon atoms, such as C_6__–1__2_ OOMs observed at Jungfraujoch [14] and C_10__–2__0_ OOMs observed at Himalaya [20].

During non-FT periods (Fig. 3c and d), OOM concentrations were generally lower. The C_4__–5_ OOM composition was very similar to those identified in FT events, but almost an order of magnitude lower in concentration. In contrast, C_6__–8_ OOMs were more varied and accounted for a larger fraction of the total OOMs than during FT events. Whereas we attribute the significantly reduced C_4__–5_ OOM concentrations to the decreased influence of FT air, the changes in C_6__–8_ OOMs appear to be due to the increased impact of BL air (or night-time residual BL air). During non-FT events, C_4__–5_ OOMs during daytime were accompanied by noticeable amounts of C_6__–8_ and C_≥9_ OOMs (Fig. 3e and f). These OOMs also contained on average more oxygen atoms than the OOMs in FT air, suggesting they had a different origin, likely oxidation products of urban biogenic and anthropogenic emissions in the nearby metropolitan area [19].

We believe that the dominant role of C_4__–5_ compounds in the observed OOMs at CHC during FT events directly reflects strong isoprene emissions from the Amazonian rainforest. During the wet season, isoprene concentrations (several ppbv (parts per billion by volume)) in the Amazon BL are approximately an order of magnitude higher than those of other BVOCs (e.g. monoterpenes (C_10_H_16_)) [21]. Shilling *et al.* reported that isoprene was still abundant (up to ∼3 ppbv) at the top of the Amazon BL (∼1-km altitude), whereas monoterpene concentrations were usually <0.2 ppbv (the instrument detection limit) [22]. Organic compounds are transported to the FT by deep convection. It is a different pathway to the BL mixing. Frequent mesoscale convective systems in the Amazon Basin [23] provide a transport mechanism for lifting organics from the BL to the tropical FT [4,5]. In this process, while organics less volatile and/or more soluble than isoprene are largely scavenged by cloud hydrometeors [24], a substantial amount of isoprene (>1 ppbv, originating from the Amazon BL) is transported to the FT over tropical South America at sunset [6].

While isoprene is short-lived when its atmospheric lifetime is determined mainly by OH oxidation during daytime (0.4 h; see ‘Methods’ for details) in the tropical FT, its lifetime is prolonged at night when O_3_ and NO_3_ radicals become its major oxidants (∼40–55 h; ‘Methods’). In contrast, the lifetime of other BVOCs, such as monoterpenes, is much shorter (3–4 h) under nocturnal FT conditions. Moreover, the primary first-generation product of OH-initiated isoprene oxidation, isoprene hydroxy hydroperoxide (ISOPOOH), has a lifetime of ∼3–5 h [25]. Further oxidation of ISOPOOH by OH leads to the formation of isoprene epoxydiols (IEPOX), the major second-generation product with a lifetime of ∼20–30 h [6,17]. The relatively longer lifetimes of isoprene (nocturnal) and its major oxidation products facilitate their long-range transport in the tropical FT compared with other BVOCs [6].

Isoprene-OOMs observed in the tropical FT are potentially the multi-generation OH-oxidation products of isoprene. The predominant CHO and CHON species in these isoprene-OOMs (Fig. 3) have been previously detected under low-NO_x_ conditions in various studies, including chamber experiments investigating isoprene OH oxidation under varying NO_x_ conditions [26] and field measurements conducted at remote, isoprene-dominated forests in Alabama, USA [19]. The observed isoprene-OOMs exhibit an overall CHON/CHO ratio of ∼1.3 : 1 (Fig. 3g), which is comparable to ratios obtained in the previous studies when NO concentrations (nitric oxide; ∼25–40 pptv (parts per trillion by volume)) were close to those observed in the tropical FT (less than ∼50 pptv) [4] and predicted by chemical transport models (10–100 pptv) [6]. Thus, isoprene-OOMs may also be prevalent in the tropical FT, leading them to be observed at CHC, located hundreds of kilometers away from the Amazon Basin.

The outflow of the mesoscale convective systems in the FT could also provide a suitable chemical environment for the formation of isoprene-OOMs observed in our study. During the study period, intensive lightning activity associated with convective systems was observed (Supplementary Figs S4 and S11), which enriches the amounts of OH and (NO) needed for forming isoprene-OOMs in this region and the Amazon FT [6,27]. OOMs previously condensed on aerosol particles in the upper part of FT can be another potential source of these isoprene-OOM vapors due to evaporation as temperature rises in descending air masses [28].

Thus, we conclude that the majority of OOMs observed in lower tropical FT at CHC were from the isoprene emitted from the Amazonian rainforest. Still, it is important to note that the chemical environment changes along the path of the air masses and the chemical transformation of OOMs may happen in the gas and particle phases. However, a detailed investigation of this mechanism is unlikely based on our mountain-top measurements.

## DISCUSSION

**Figure 4.**
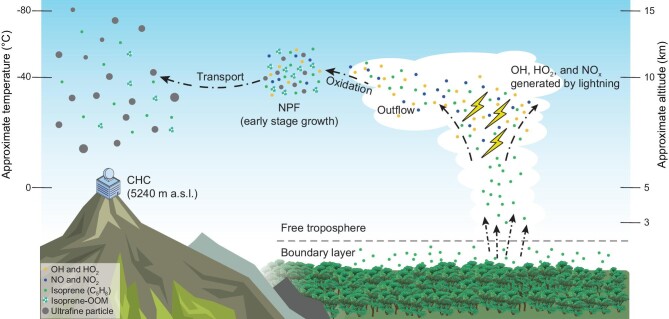
Life cycle of isoprene-OOMs and their role in NPF over the tropical FT. Isoprene emitted from the Amazon rainforest is transported upwards by mesoscale convective systems (convective clouds) to the tropical FT (right). Isoprene surviving from removal processes in the clouds (e.g. scavenging by cloud hydrometeors) reacts with hydroxyl radicals (OH), hydroperoxyl radicals (HO_2_) and nitrogen oxides (NO and NO_2_) produced by lightning in the convective clouds [27]. In the cloud outflow, isoprene-OOMs (C_4__–5_ CHOs and C_4__–5_ CHONs) are formed. These OOMs can contribute to NPF in the tropical FT, particularly the growth of newly formed nanoparticles (middle). The isoprene-OOMs, which can endure long-range transport in the lower and middle regions of tropical FT, are potentially involved in aerosol formation on a continental scale (left).

The question is whether the observed isoprene-OOMs play a role in NPF to form large numbers of UFPs observed over the tropical FT (Fig. 4). Previous model studies have concluded that OOMs play a crucial role in NPF in the tropical FT, contributing to a significant portion of CCN in this region [7,8]. The NPF parameterizations utilized in these studies predominantly relied on OOMs extracted from chamber experiments, which investigated NPF driven by monoterpene-derived OOMs [29,30]. In contrast, isoprene-OOMs usually show a suppressive effect on NPF in chamber studies [31–33] and are likely the reason for NPF to be rarely observed in the Amazon BL [3,4]. However, the potentially widespread isoprene-OOMs in the tropical FT may exert a spatially broader influence on aerosol formation compared with non-isoprene-OOMs. Furthermore, isoprene-OOMs measured by using the nitrate-based CI-APi-TOF constitute a relatively small fraction of the total isoprene oxidation products. While the majority of the oxidation products are composed of less oxygenated and more volatile species (e.g. ISOPOOH and IEPOX) than isoprene-OOMs [34,35], they may still contribute to particle growth in the tropical FT by condensing on larger-sized particles [6]. Isoprene-OOMs formed through multi-generation oxidation of isoprene have higher oxidation states than other oxidation products and thus may participate in NPF more efficiently due to their lower volatility (see [36] and ‘Methods’).

In addition to the gas-phase observations during the wet season, enhanced contributions of C_4__–5_ OOMs were observed in the particle phase at CHC when particle chemical composition measurements were available (in April 2018 during the wet-to-dry transition period; ‘Methods’). On the night of 22 April 2018, CHC was affected by FT air from the western part of Amazonia (Supplementary Fig. S12). As in the winter FT events, the observed particles were predominated by small Aitken-mode particles with decreasing WVMR and eBC concentrations. Concentrations of gaseous C_4__–5_ OOMs increased while C_6__–8_ and C_≥9_ OOMs decayed during that time (from 19 : 00 to 21 : 00). The mass fraction of isoprene-OOMs determined from the gas-phase and particle-phase measurements increased almost concurrently (as shown in Supplementary Fig. S12e). Compared with particles within the BL air, a more evident contribution of isoprene-OOMs was determined in the observed particles from the FT air. Nevertheless, the potential contribution from larger particles to the increased mass fraction of isoprene-OOMs cannot be completely ruled out. These particles might account for a larger mass fraction than Aitken-mode particles in the FT air, even though their number concentrations were much lower.

While a complete NPF event in the tropical FT (i.e. both nucleation and the growth of newly formed nanoparticles) has not yet been directly observed at CHC and in the aircraft-based aerosol studies [1,3,37], the rapid increase in UFPs is clearly attributed to recently occurred NPF in the FT [1,3]. The small sizes of these nanoparticles indicate that they were newly formed in the tropical FT; otherwise, they would have soon grown to larger sizes during transport. Thus, higher concentrations of isoprene-OOMs and their enhanced contributions in the particle-phase measurements suggest that these OOMs play a potentially important role in tropical FT NPF, particularly in the growth of newly formed nanoparticles.

Moreover, the saturation vapor pressure of isoprene-OOMs may reduce to a sufficiently low level in the upper part of tropical FT, where temperatures are extremely low (e.g. approximately –40°C at 10 km a.s.l. [4]). As a result, it is plausible that isoprene-OOMs have a chance to participate in aerosol nucleation in the FT [8], such as through H_2_SO_4_–OOMs nucleation and/or pure biogenic nucleation. However, their role in aerosol nucleation remains speculative and requires further observational evidence, such as direct observation of the aerosol nucleation process in the upper tropical FT. Nevertheless, the observed isoprene-OOMs are important constraints for NPF parameterizations in chemical transport models, adding more value to future studies simulating aerosol formation in the tropical FT.

We present a comprehensive analysis of organic trace gases and atmospheric aerosol *in situ* observations at CHC in Bolivia—one of the highest atmospheric observatories in the world. Mountain-top state-of-the-art mass spectrometry [9], combined with detailed, highly resolved air mass analyses [10], demonstrates the presence of OOMs in both gas and particle phases in tropical FT air from Amazonia. Our results indicate that the observed OOMs are dominated by oxidation products of isoprene emitted from the rainforest >800 km away. In the tropical FT, these isoprene-OOMs can contribute to NPF, at least the growth of newly formed nanoparticles, on a continental scale. Such molecular-level observations of OOMs are unprecedented for the tropical FT, providing a crucial step toward understanding aerosol formation over the tropics.

## METHODS

### CHC station

The GAW station CHC (16.3505 S, 68.1314 W, Extended Data Fig. 1) is located at 5240 m a.s.l. near the summit of Mount Chacaltaya in the Bolivian Andes. It is ∼17 km north and ∼1.6 km above the La Paz—El Alto metropolitan area, which has a population of ∼1.7 million, and close to the Bolivian Amazonia (including Beni, Santa Cruz, north of La Paz departments). A detailed description of the station and its surrounding area can be found on the website of the CHC station (http://www.chacaltaya.edu.bo/) and in the studies conducted at CHC [9,11,12,38,39].

### SALTENA campaign

The SALTENA campaign was conducted from December 2017 to May 2018, aiming to understand the formation/growth mechanism and properties of aerosols measured at CHC. The 6-month measurement campaign was arranged in order to cover the wet season (December to ∼February), transition period (∼March to April) and dry season (May). Our study mainly focuses on measurements during the wet season in January 2018, when CHC was significantly affected by Amazon FT air [10], and OOM measurement data were continuously available (i.e. from 6 to 22 January 2018).

It is worth mentioning that more frequent and intensive NPF events were observed in the daytime BL during the dry season at CHC, likely driven by H_2_SO_4_–NH_3_ cluster ions [38,39]. This is due to a shift in the predominant air mass origins between the wet and dry seasons. During the wet season, air sampled at CHC originates from the Amazon Basin, located east of CHC. In contrast, during the dry season, the air mass arriving at CHC is mainly from the Altiplano region, located west of CHC, where active volcanic degassing of SO_2_ has been observed. However, we cannot completely rule out the potential contribution of organic compounds to the NPF in the daytime BL at CHC. Further details on dry-season NPF can be found in a recent paper by Zha *et al.*, which is based on cluster ion and aerosol measurements from the same measurement campaign [39].

### Instrumentation

#### APi-TOF

The APi-TOF (TOFWERK AG and Aerodyne Research) was deployed to measure the chemical composition of naturally charged negative ions in January 2018 at CHC. The APi allows the instrument to sample ions in ambient air directly by reducing the pressure of the sampled airflow (14 standard liters per minute (SLPM) in total and 0.8 SLPM go into the instrument) from atmospheric pressure to ∼10^−4^ mbar. The ions are focused and guided by two quadrupoles and an ion lens in the APi before entering the time-of-flight mass spectrometer (TOF-MS; ∼10^−6^ mbar). In this part, ions are detected and identified. The resolving power was ∼5000 Th/Th. A description with more details of this instrument is presented in Junninen *et al.* [40]. APi-TOF data used in this study were averaged to 1-h resolution.

#### CI-APi-TOF

The CI-APi-TOF (TOFWERK AG and Aerodyne Research) is an APi-TOF coupled with a CI unit using nitric acid (HNO_3_) as the ionization reagent. The instrument is extensively used to measure oxidized organic compounds and sulfuric acid (H_2_SO_4_) [41]. In the CI module, the nitrate ion is generated by exposing the sheath flow (20 SLPM) that contains HNO_3_ to soft x-ray radiation and then charging the neutral molecules in the sampling airflow (10 SLPM) within a reaction time of ∼200 ms before they enter the APi and the TOF-MS modules. The instrument was calibrated with H_2_SO_4_ using the set-up described in Jokinen *et al.* [41]. After including the diffusion loss of H_2_SO_4_ in the 1.5-m sampling line, a calibration factor of 1.5 × 10^10^ molecules cm^−3^ was obtained and used for determining the concentration of OOMs.

The OOMs concentrations were estimated in two steps. We first corrected the measured signal intensity with a mass-dependent transmission function that depends on the setting of the instrument and was determined by using the method described in a previous study [42]. After the transmission correction, the calibration factor of H_2_SO_4_ was used to estimate the observed OOMs concentrations. It is important to note that OOMs might not be charged as efficiently as H_2_SO_4_ by nitrate ions (NO_3_^−^) in the CI unit [43]. As a result, OOMs concentrations presented in this study could be underestimated. A lower detection limit of ∼5 × 10^4^ molecule cm^−3^ was determined from H_2_SO_4_ and zero measurements. The CI-APi-TOF data included in this study were averaged to 10-min resolution.

#### FIGAERO HR-TOF-CIMS

The filter inlet for gases and aerosols (FIGAERO) coupled to a high-resolution time-of-flight chemical ionization mass spectrometer (FIGAERO HR-TOF-CIMS, Aerodyne Research) using iodide (I^–^) as the reagent ion was deployed to measure the molecular composition of organic compounds and inorganic acids. The FIGAERO inlet can be operated in gas-phase and particle-phase modes. In the gas-phase mode, ambient air is directly sampled into the ion-molecule reactor while particles are simultaneously collected to a polytetrafluoroethylene filter through another sampling port. In the particle-phase mode, a nitrogen gas stream is heated and blown through the filter to evaporate the particles via temperature-programmed desorption. More details about the instrument can be found in Mohr *et al.* [44]. It is important to note that the FIGAERO HR-TOF-CIMS was only deployed from April (transition season) at CHC and no measurement was available in January 2018 during our study period (wet season).

#### MPSS

The mobility particle size spectrometer (MPSS) was deployed to measure particle number size distribution in a size range of 10–500 nm at CHC [45]. The instrument consists of a bipolar diffusion charger, a Hauke-type differential mobility analyser and a TSI 3772 condensation particle counter.

#### MAAP

The multi-angle absorption photometer (MAAP, Thermo-Scientific model 5012) was used to determine eBC mass concentrations at CHC [46]. The lower detection limit of this instrument is ∼0.05 μg m^−3^ at a time resolution of 10 min.

#### Ancillary measurements

Air temperature, relative humidity (with respect to water) and atmospheric pressure were measured using an automatic weather station at CHC.

### Model simulation

#### WRF simulation

The weather research and forecasting (WRF) model is an advanced non-hydrostatic numerical weather prediction model [47] that can reproduce meteorological situations at a wide range of spatial and temporal scales. In this study, WRF version 4.0.3 was used. Four nested domains were used with grid spacings ranging from 38 km (the outmost domain, with the Amazon Basin, tropical Andes and west Pacific ocean included) to 1 km (the innermost domain, including complex mountainous topography surrounding CHC and the interface between the Andes and the Amazon Basin included). A detailed description of the model set-up and parameterization is given in Aliaga *et al.* [10].

#### FLEXPART simulation

The 96-hour history and footprint of air masses arriving at CHC were determined using the flexible particle dispersion model (FLEXPART). Different versions of the FLEXPART model have been developed to adapt to a range of numerical weather prediction models. In this study, we used the latest version of the FLEXPART-WRF model (version 3.3.2) [48] driven by the high-resolution meteorological output from the WRF simulation. In this way, more validity and accuracy were added to the FLEXPART air mass history simulation and made this simulation state-of-the-art compared with other similar studies in the southern hemisphere tropics of South America.

In the FLEXPART simulations, we continuously release 20 000 particles per hour from the 0- to 10-m layer above ground level (a.g.l.) at CHC. The output of the FLEXPART-WRF, when running in backward mode, is a SRR. The SRR is the aggregated residence time of the passive air tracer particles at each 3D grid cell. The values are normalized by the total number of particles such that if all of the 20 000 particles were to reside in only one cell, the SRR values of this cell would equal 96 h (i.e. the total backward simulation time). The derived SRRs describe the relationship between each 3D grid cell in the simulation (as potential source regions) and the air masses arriving at the measurement station (receptor). In general, a higher SRR of a grid cell indicates a larger contribution to the observed air masses. The detailed model set-up is also described in Aliaga *et al.* [10].

### Estimation of the WVMR

The WVMR is a measure of the mass concentration of water vapor in the atmosphere, which is calculated as follows:


(1) \begin{eqnarray*}
{\textit{WVMR}}\ = \ B \times \frac{{{P}_{\rm w}}}{{{P}_{\rm tot} - {P}_{\rm w}}},
\end{eqnarray*}

where *B* is a constant (621.9907 g kg^−1^, molecular weight ratio of water to dry air); *P_w_* and *P_tot_* are the water vapor pressure and the atmospheric pressure, respectively. In this study, *P_w_* was determined based on the method presented in Buck *et al.* using the ambient temperature, relative humidity (RH) and pressure measured at CHC [49].

### Identification of FT events

The periods when FT air dominated at CHC were identified as FT events, as mentioned in the main text. Air mass history analysis shows that, on average, FT air constituted ∼70% of air masses arriving at CHC in January 2018. However, the observed air masses were usually not purely from the FT due to the concurrent local influence on CHC, such as BL air from the nearby La Paz—El Alto metropolitan area. The impact of BL air at CHC increases with the effect of thermally driven local air circulation during daytime (07 : 00–18 : 00; all the times are in local time, UTC–4) [9–12]. This is indicated by the advecting eBC concentration at noontime at CHC [11]. Therefore, to minimize the influence of the BL on CHC, we only used the measurements during night-time (19 : 00–06 : 00) [12].

The presence of water in the atmosphere (measured by e.g. WVMR and RH) and black carbon were used to identify the influence of FT air in the previous studies [3,50]. This is due to FT air in the tropics typically containing much less water and being much cleaner (biomass burning is inhibited by high-level rainfall and moisture during the wet season [51]) than BL air [12,13,52]. Thus, to characterize the influence of FT air, WVMR and eBC concentrations were introduced as further parameters to identify FT events at CHC. Thresholds were 5.5 g kg^−1^ in the WVMR (denotes the lower 30% of the night-time WVMR during the study period) and 0.08 μg m^−3^ in the eBC concentration (eBC night-time mean concentration during the study period) were used.

### Estimation of atmospheric lifetimes of BVOCs

The atmospheric lifetimes of isoprene (τ_isoprene_) and monoterpene (τ_monoterpene_) against different oxidants (i.e. OH, O_3_ and NO_3_ radical) are roughly estimated using the temperature-dependent rate coefficient recommended by the International Union of Pure and Applied Chemistry (IUPAC) under the mid-to-upper tropical FT condition (6-km altitude). The air temperature (268 K), pressure (0.44 atm) and concentrations of OH and O_3_ (0.5 and 70 pptv, respectively) in this region are adapted from Liu *et al.* [8]. The concentration of NO_3_ radical (1 pptv) is extracted from Brown *et al.* [53].

The rate coefficients between isoprene and OH, O_3_ and NO_3_ are (2.7 × 10^−11^)e^(390/T)^, (1.03 × 10^−14^)e^(^^–1^^995/T)^ and (3.15 × 10^−12^)e^(^^–4^^50/T)^ cm^3^ molecule^−1^ s^−1^ [54]. The corresponding τ_isoprene_ against OH, O_3_ and NO_3_ are 0.4, 55.4 and 39.8 h, respectively. In contrast, the rate coefficients between α-pinene, the most abundant monoterpene above the Amazon rainforest [55], and OH, O_3_ and NO_3_ are (1.2 × 10^−11^)e^(440/T)^, (6.3 × 10^−16^)e^(^^–5^^80/T)^ and (1.2 × 10^−12^)e^(490/T)^ cm^3^ molecule^−1^ s^−1^ [54]. The corresponding τ_α-pinene_ against OH, O_3_ and NO_3_ are 0.8, 4.6 and 3.1 h, respectively.

## Supplementary Material

nwad138_Supplemental_File

## Data Availability

Data for all figures in the main text are available at the public data repository (https://doi.org/10.5281/zenodo.7908052).
