# Supplementary material for: Oxidized organic molecules in the tropical free troposphere over Amazonia
Source: Natl Sci Rev. 2023 May 15;11(1):nwad138. doi: 10.1093/nsr/nwad138 (PMC10727843; doi:10.1093/nsr/nwad138)
Supplement: nwad138_Supplemental_File [file nwad138_supplemental_file.docx]

# Supplementary file for “Oxidized organic molecules in the tropical free troposphere over Amazonia”

Qiaozhi Zha^1,2^, Diego Aliaga^1^, Radovan Krejci^3^, Victoria Sinclair^1^, Cheng Wu^3^, Giancarlo Ciarelli^1^, Wiebke Scholz^4^, Liine Heikkinen^1,3^, Eva Partoll^4^, Yvette Gramlich^3^, Wei Huang^1^, Markus Leiminger^4,5^, Joonas Enroth^1^, Otso Peräkylä^1^, Runlong Cai^1^, Xuemeng Chen^1^, Alkuin Maximilian Koenig^6^, Fernando Velarde^6^, Isabel Moreno^6^, Tuukka Petäjä^1^, Paulo Artaxo^7^, Paolo Laj^1,8^, Armin Hansel^4^, Samara Carbone^9^, Markku Kulmala^1,2,10^, Marcos Andrade^5,11^, Douglas Worsnop^1,12^, Claudia Mohr^2^, Federico Bianchi^1,*^

^1^ Institute for Atmospheric and Earth System Research / Physics, University of Helsinki, Helsinki, 00014, Finland

^2^ Joint International Research Laboratory of Atmospheric and Earth System Sciences, Nanjing University, Nanjing 210023, China

^3^ Department of Environmental Science & Bolin Centre for Climate Research, Stockholm University, Stockholm, SE-106 91, Sweden

^4^ Institute for Ion and Applied Physics, University of Innsbruck, Innsbruck 6020, Austria

^5^ Ionicon Analytik GmbH, Innsbruck, 6020, Austria

^6^ Laboratory for Atmospheric Physics, Institute for Physics Research, Universidad Mayor de San Andrés, La Paz, Bolivia

^7^ Institute of Physics, University of Sao Paulo, Sao Paulo, 05508-900, Brazil

^8^ Institute for Geosciences and Environmental Research (IGE), University of Grenoble Alpes, Grenoble, 38000, France

^9^ Agrarian Sciences Institute, Federal University of Uberlândia, Uberlândia, 38408-100, Brazil

^10^ Beijing Advanced Innovation Center for Soft Matter Science and Engineering, Beijing University of Chemical Technology, Beijing, 100029, China

^11^ Department of Atmospheric and Oceanic Sciences, University of Maryland, College Park, MD 20742, USA

^l2^ Aerodyne Research, Inc., Billerica, MA 01821, USA

*Corresponding author. Email: [federico.bianchi@helsinki.fi](mailto:federico.bianchi@helsinki.fi)

**Table S1** List of major OOMs that were identified with nitrate CI-APi-TOF during FT events. The reagent ion (NO_3_^-^) is not included in the chemical formula of OOMs.

| Chemical formula | m/z (Th) |
| --- | --- |
| C_4_H_6_O_4_ | 118.0266 |
| C_4_H_8_O_4_ | 120.0422 |
| C_5_H_6_O_4_ | 130.0266 |
| C_5_H_8_O_4_ | 132.0426 |
| C_4_H_6_O_5_ | 134.0215 |
| C_4_H_8_O_5_ | 136.0372 |
| C_5_H_6_O_5_ | 146.0215 |
| C_5_H_8_O_5_ | 148.0372 |
| C_4_H_7_O_3_(ONO_2_) | 149.0324 |
| C_5_H_10_O_5_ | 150.0528 |
| C_4_H_9_O_3_(ONO_2_) | 151.048 |
| C_5_H_7_O_3_(ONO_2_) | 161.0324 |
| C_5_H_9_O_3_(ONO_2_) | 163.0481 |
| C_4_H_7_O_4_(ONO_2_) | 165.0273 |
| C_4_H_9_O_4_(ONO_2_) | 167.043 |
| C_8_H_12_O_4_ | 172.0736 |
| C_5_H_7_O_4_(ONO_2_) | 177.0273 |
| C_6_H_10_O_6_ | 178.0477 |
| C_5_H_9_O_4_(ONO_2_) | 179.043 |
| C_8_H_14_O_6_ | 206.079 |
| C_7_H_9_O_5_(ONO_2_) | 219.0379 |
| C_7_H_11_O_5_(ONO_2_) | 221.0536 |


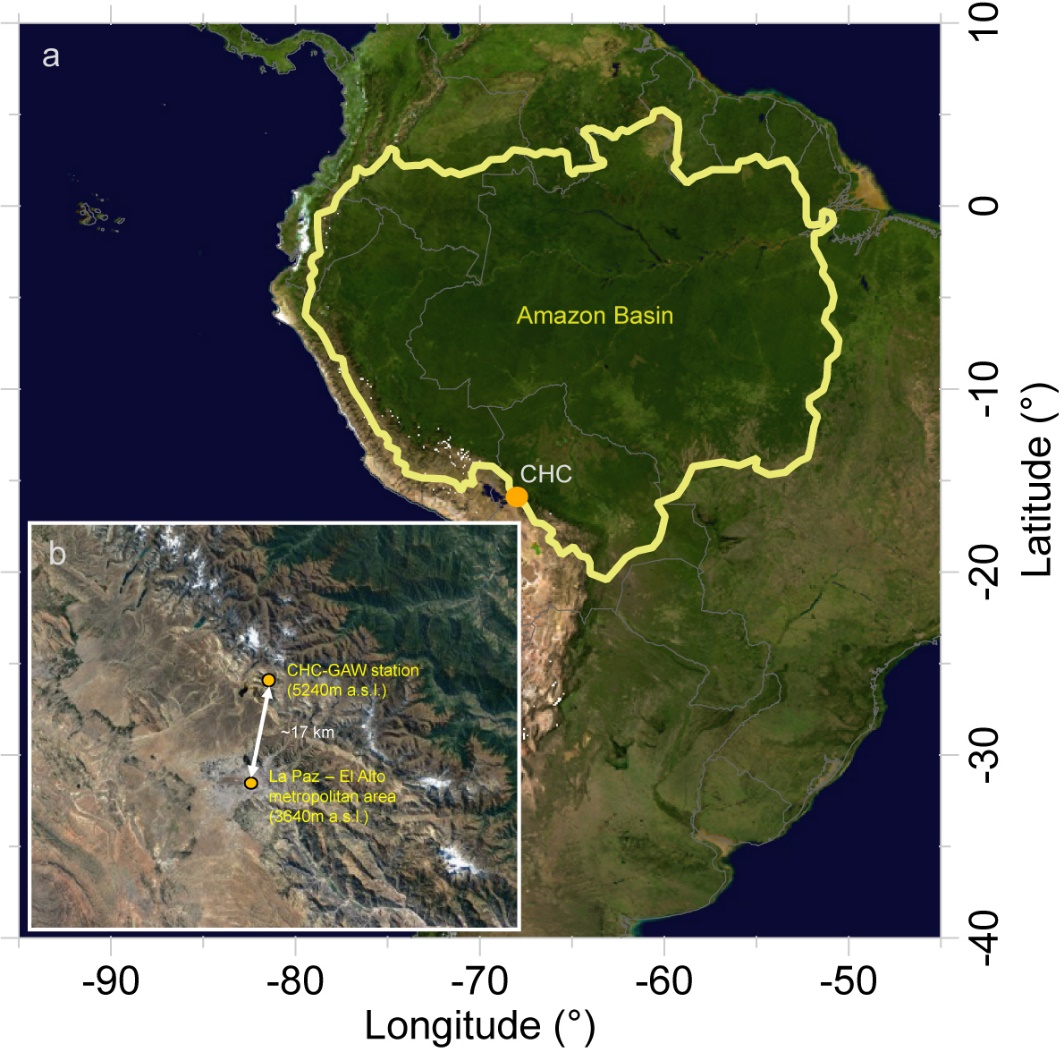


**Figure S1. a**, Google Earth satellite images showing locations of CHC, **b**, La Paz- El Alto metropolitan area. The yellow outline denotes the borders of the Amazon Basins (Charity et al., 2016).


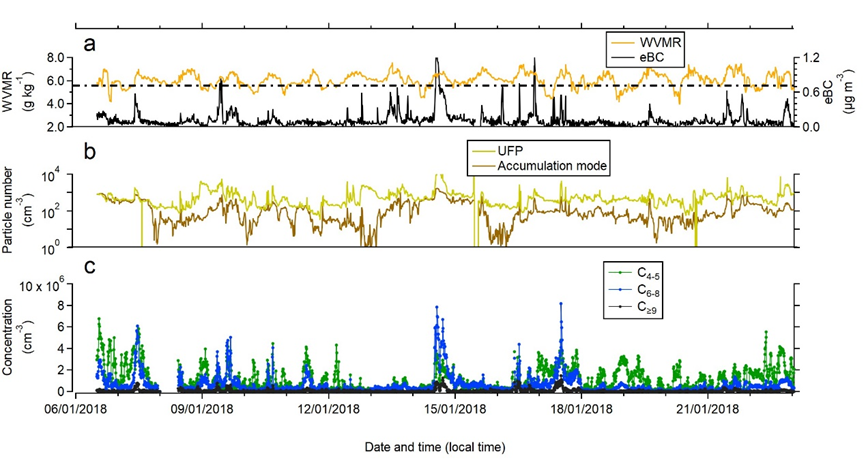


**Figure S2.** Time series of **a**, WVMR and eBC concentrations, **b,** UFPs and accumulation mode particles, and **c,** C_4-5_, C_6-8_, and C_≥9_ OOMs from 6 to 22 January 2018. The dashed line in panel a indicates one of the thresholds for identifying FT events (WVMR ≤ 5.5 g kg^-1^).


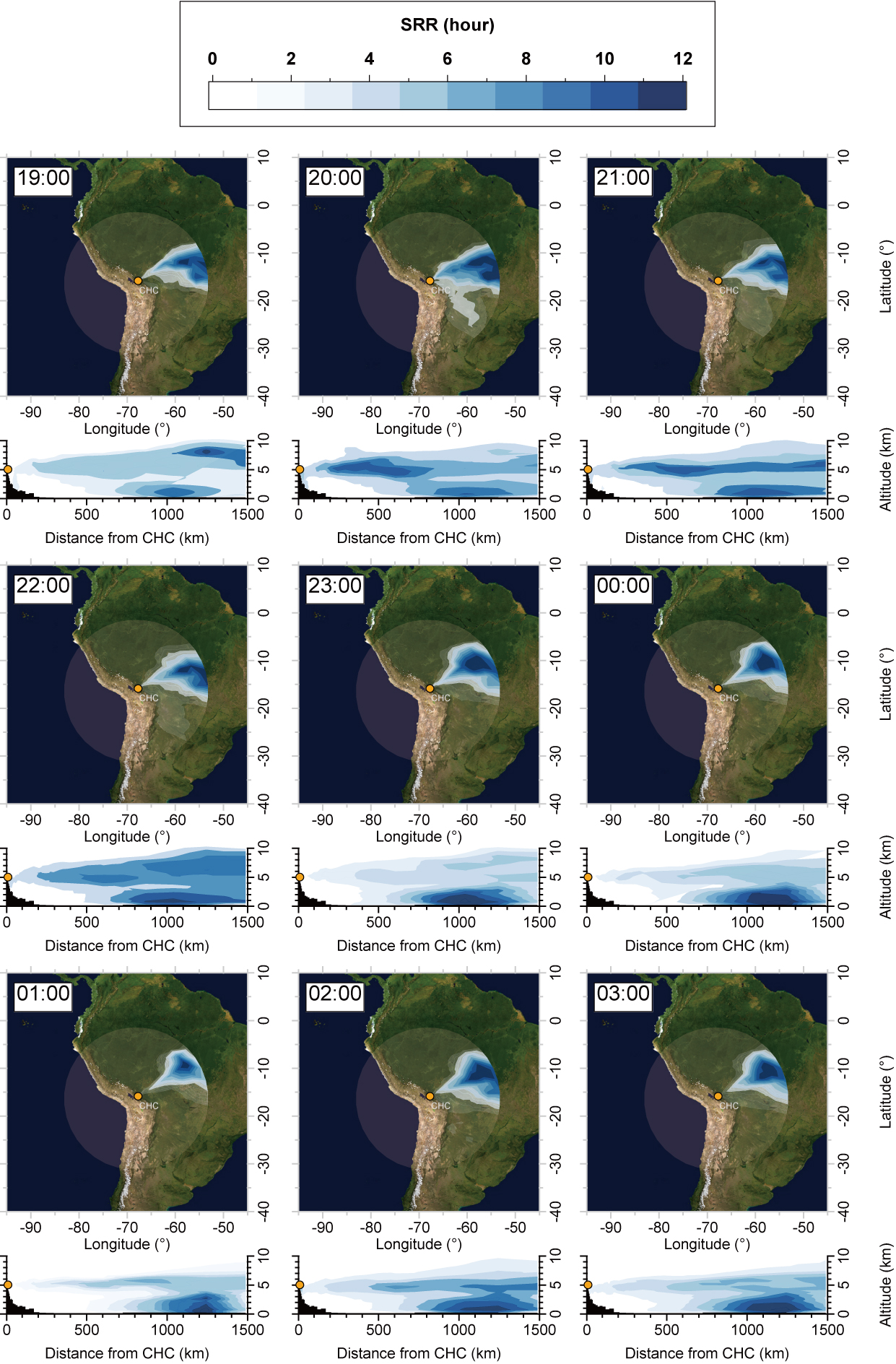


**Figure S3.** 96-hour WRF-FLEXPART air mass history and footprint for each hour from 19:00 – 03:00 on the night of 10 January 2018. The color bar denotes the SRR of the passive air tracers integrated in the radial direction. The black shaded area indicates the topography near the station. The lower SRR intensities in the region close to CHC are likely due to the short residence time of the passive air tracers. The semi-transparent circle indicates the horizontal output domain of the model.


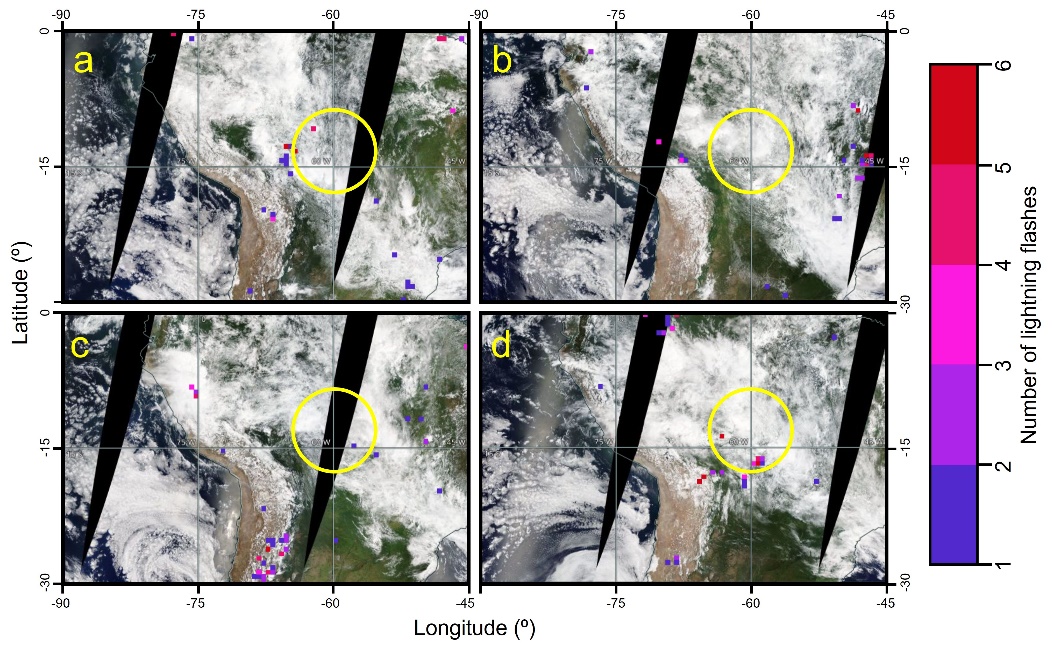


**Figure S4.** True-color daily satellite images from Terra/MODIS corrected reflectance imagery (Gumley et al., 2010) and daily lightning flash counts International Space Station (ISS) Lightning Imaging Sensor (LIS) data (Blakeslee et al., 2021) on **a**, 10 January 2018, **b**, 9 January 2018, **c**, 8 January 2018, and **d**, 7 January 2018. The yellow circle in each panel is a rough indication of the region where the SRRs have the highest intensities in Fig. 1.


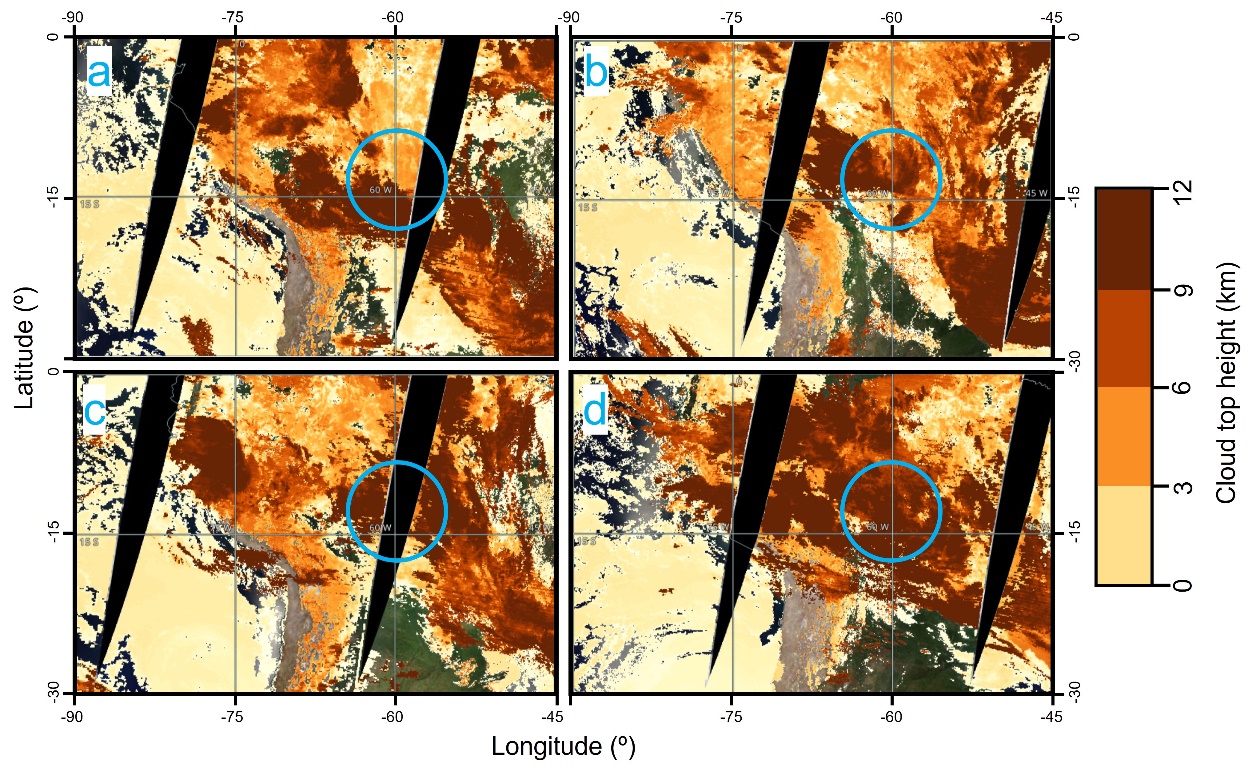


**Figure S5.** Cloud top height satellite images from Terra/MODIS on **a**, 10 January 2018, **b**, 9 January 2018, **c**, 8 January 2018, and **d**, 7 January 2018. The blue circle in each panel is a rough indication of the region where the SRRs have the highest intensities in Fig. 1.


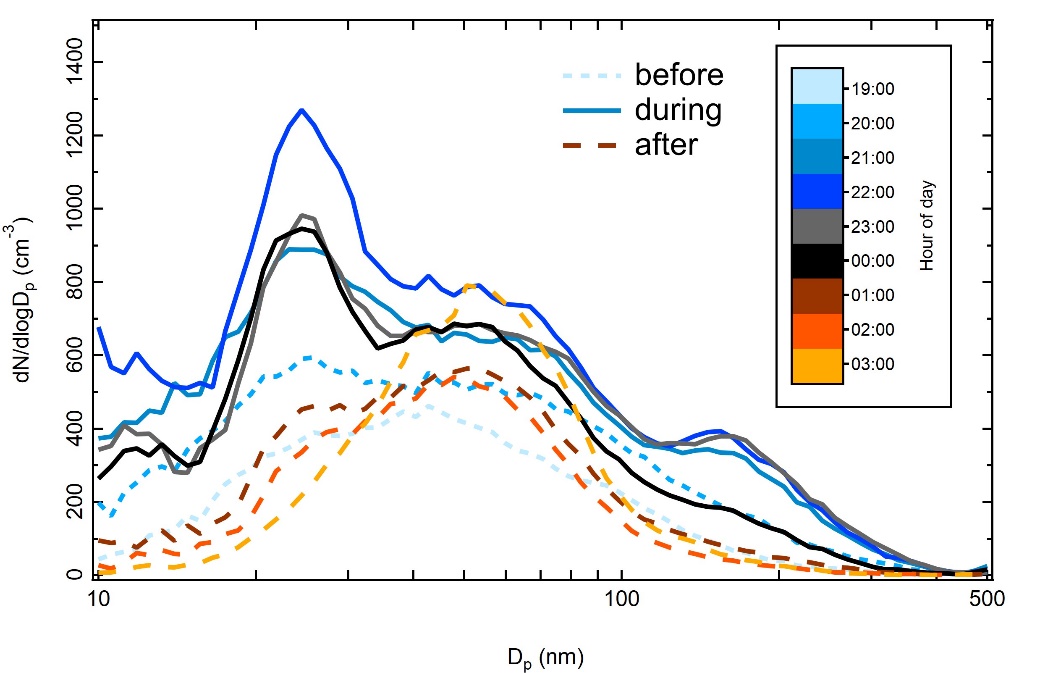


**Figure S6.** Evolution of the hourly averaged particle number size distribution during the night of 10 January 2018. Dashed, solid, and dotted lines denote particle size spectra observed before (19:00 – 20:00), during (21:00 – 00:00), and after (01:00 – 03:00) the FT event, respectively. It is noted that the influence of FT air on the observed aerosol particles at CHC is already evident at 20:00. The concurrent increases in accumulation mode particles indicated that a fraction of the UFPs originating in the Amazon FT could have grown to larger sizes during the transport to CHC (takes ~2 days; Froyd et al., 2009).


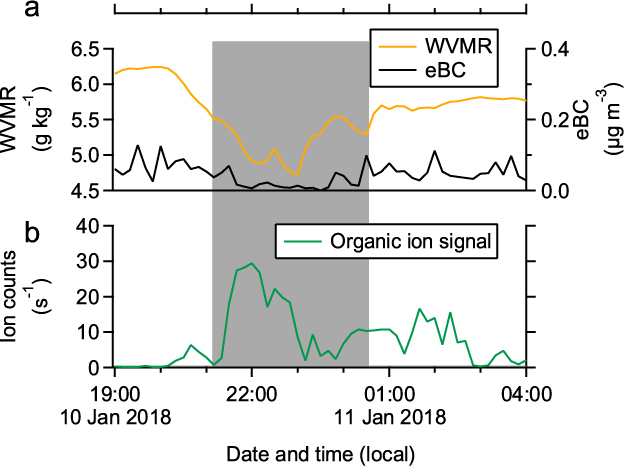


**Figure S7.** The Amazon FT event observed at CHC during the night of 10 January 2018 (as shown in Fig. 1). **a**, Concentrations of the water vapor mixing ratio (WVMR) and eBC at CHC. The grey shaded area denotes the FT event period (from ~21:00 to ~01:00, WVMR ≤5.5 g kg-1 and eBC ≤0.08 µg m-3) at CHC. **b**, Signal of organic ions measured with the APi-TOF.


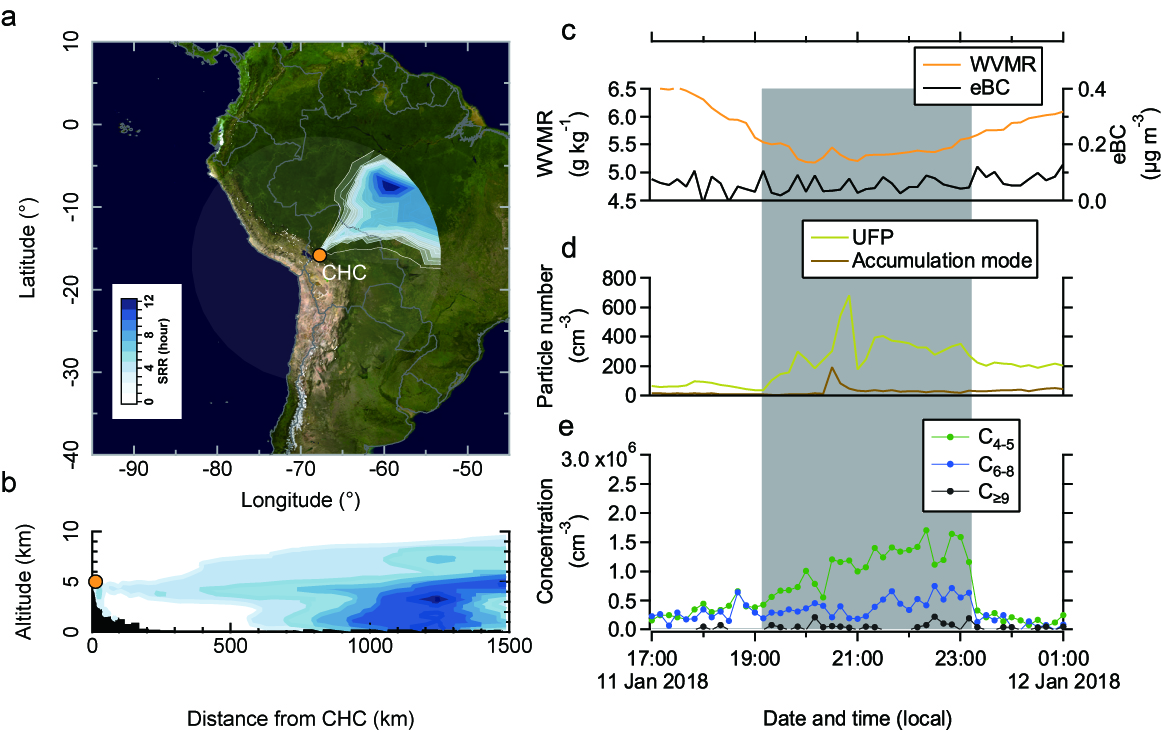


**Figure S8.** An Amazon FT event observed at CHC during the night of 11 January 2018. **a**, Map and horizontal profile of the vertically integrated SRR averaged from 19:00 to 23:00. The color bar denotes the SRR of the passive air tracers. **b**, Vertical profile of the SRR integrated in the radial direction and averaged from 19:00 to 23:00. The black shaded area indicates the topography condition near the station. **c**, Concentrations of WVMR and eBC. **d**, Concentrations of UFPs and accumulation mode particles. **e**, Concentrations of C_4-5_, C_6-8_, and C_≥9_ OOMs. The grey shaded area denotes the FT event period (from 19:10 to 23:20) at CHC. Note that the scales of y-axis in the c, d, and e panels may be different from those in Fig. 1.


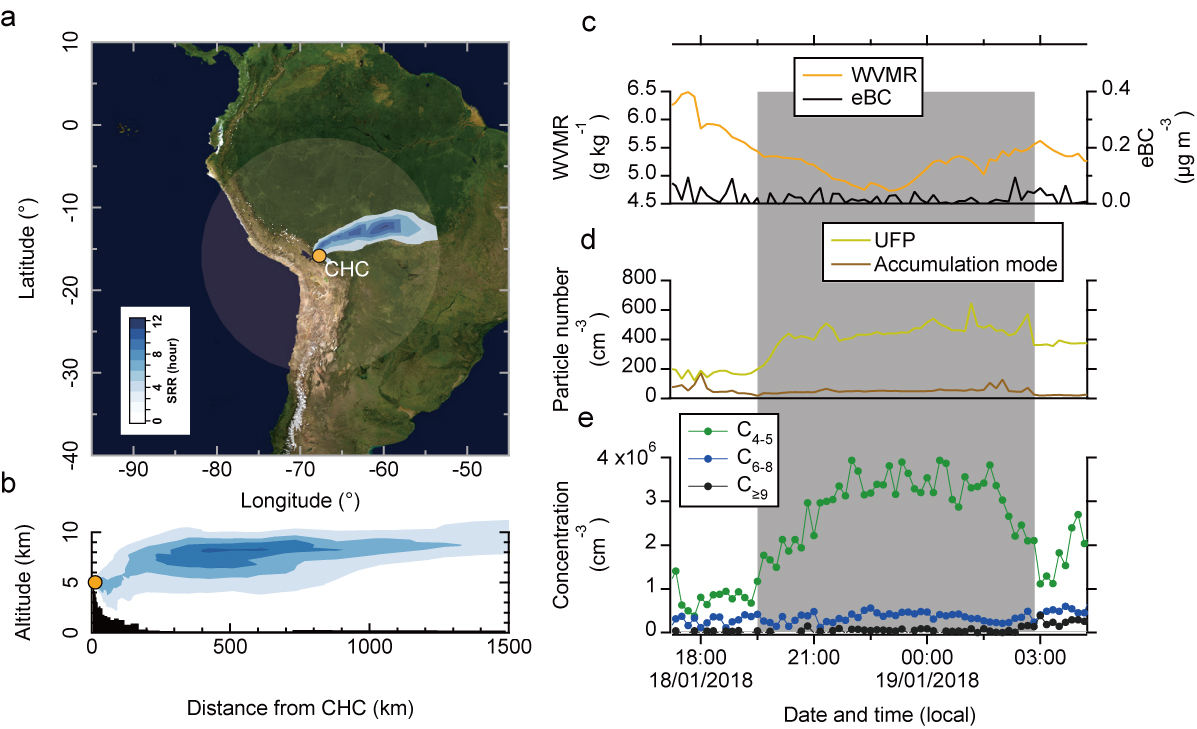


**Figure S9.** An Amazon FT event observed at CHC during the night of 18 January 2018. **a**, Map and horizontal profile of the vertically integrated SRR averaged from 20:00 to 03:00. The color bar denotes the SRR of the passive air tracers. **b**, Vertical profile of the SRR integrated in the radial direction and averaged from 20:00 to 03:00. The black shaded area indicates the topography condition near the station. **c**, Concentrations of WVMR and eBC. **d**, Concentrations of UFPs and accumulation mode particles. **e**, Concentrations of C_4-5_, C_6-8_, and C_≥9_ OOMs. The grey shaded area denotes the FT event period (from 19:30 to 02:50) at CHC. Note that the scales of y-axis in the c, d, and e panels may be different from those in Fig. 1. The convection process in this FT event is not fully captured in the air mass history analysis, like-ly due to the lower strength and/or smaller scale compared to the events on 10 and 11 January 2018.


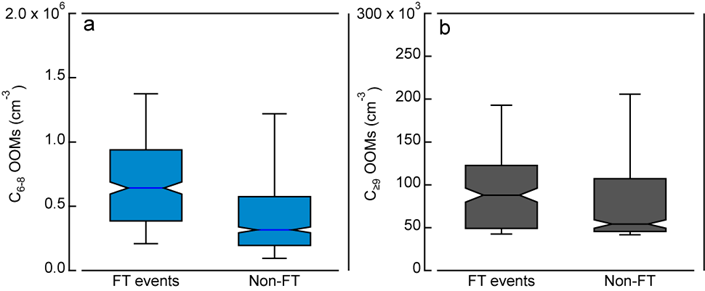


**Figure S10.** Variation in the parameters measured during periods with FT and non-FT events. **a**, Concentrations of C_6-8_ OOMs. **b**, Concentrations of C_≥9_ OOMs. This figure shows the same data as Fig. 3f and 3g but with the y-axis on smaller scales. Concentrations of C_≥9_ OOMs were close to the detection limit during the study period. Boxes and whiskers are plotted for the 10th, 25th, 50th, 75th, and 90th percentiles. Notches denote the 95% confidence interval of the median value. The number of data points (10-minute resolution) for FT events and non-FT periods are 370 and 842, respectively.


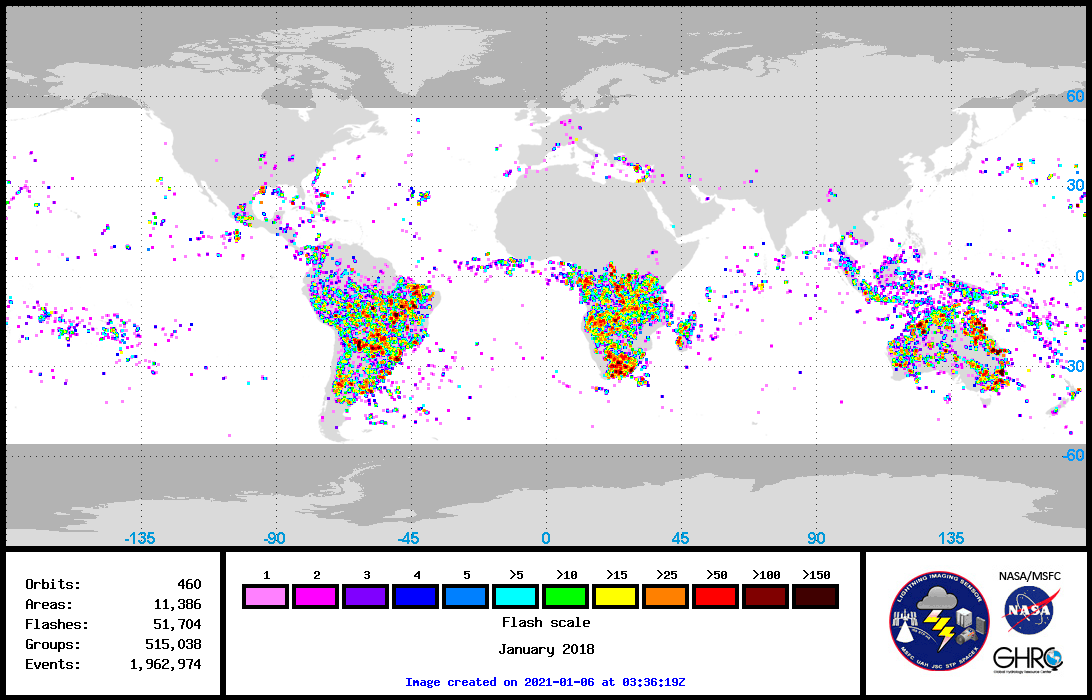


**Figure S11.** Global lightning activity distribution in January 2018 from the ISS LIS dataset (Andreae et al., 2018).


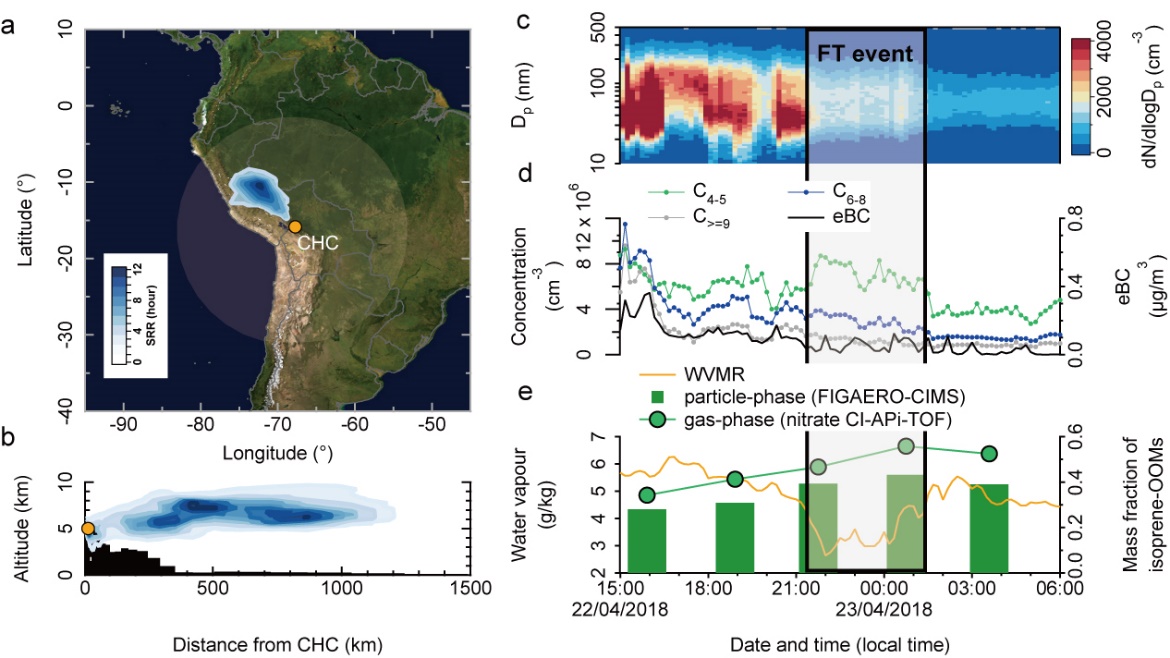


**Figure S12.** An Amazon FT event observed at CHC during the night of 22 April 2018. **a**, Map and horizontal profile of the vertically integrated SRR averaged from 21:00 to 02:00. **b**, Vertical profile of the SRR integrated in the radial direction and averaged from 21:00 to 02:00. The color bar denotes the SRR of the passive air tracers. The black shaded area in panel b denotes the topography condition near the station. **c**, Particle number size distribution (dN/dlogDp) in 10-minute time resolution. **d**, Concentrations of gas-phase C_4-5_, C_6-8_, and C_≥9_ OOMs and eBC. **e**, Concentrations of the water vapor mixing ratio (WVMR) and mass fraction of the isoprene-OOMs determined in gas-phase and particle-phase measurements with nitrate CI-APi-TOF and FIGAERO-CIMS, respectively. It is important to note that isoprene-OOMs included in the mass fraction calculation are C_4-5_ OOMs observed by nitrate CI-APi-TOF. The mass fraction of gas-phase isoprene-OOMs is calculated over the same time resolution as particle-phase measurements. The grey shaded area in panels c and d denotes the period in which the FT event was observed.

**Supplemental References**

Charity S, Dudley N and Oliveira D *et al.* Living Amazon Report 2016: A regional approach to conservation in the Amazon. WWF Living Amazon Initiative Brasília and Quito; 2016.

Gumley L. Creating Reprojected True Color MODIS Images: A Tutorial. Space Science Engineering Center University Wisconsin-Madison; 2010.

Blakeslee RJ. NRT Lightning Imaging Sensor (LIS) on International Space Station (ISS) Science Data (2021); doi: 10.5067/LIS/ISSLIS/DATA109.

Froyd KD, Murphy MD and Sanford TJ *et al.* Aerosol composition of the tropical upper troposphere. *Atmos Chem Phys* 2009; **9**: 4363–4385.

Andreae MO, Afchine A, and Albrecht R *et al.* Aerosol characteristics and particle production in the upper troposphere over the Amazon Basin. *Atmos Chem Phys* 2018; **18**: 921–961.
